# Al_2_O_3_ Coatings on Magnesium Alloy Deposited by the Fluidized Bed (FB) Technique

**DOI:** 10.3390/ma11010094

**Published:** 2018-01-09

**Authors:** Gabriele Baiocco, Gianluca Rubino, Vincenzo Tagliaferri, Nadia Ucciardello

**Affiliations:** 1 Dipartimento di Ingegneria dell’Impresa “Mario Lucertini”, University of Rome of Tor Vergata, Via del Politecnico 1, 00133 Rome, Italy; gabriele.baiocco@uniroma2.it (G.B.); tagliafe@uniroma2.it (V.T.); 2 Dipartimento di Economia ed Impresa, Univesità degli Studi della Tuscia, Via del Paradiso 47, 01100 Viterbo, Italy; gianluca.rubino@unitus.it

**Keywords:** magnesium alloy, fluidized bed, functional coating, corrosion resistance

## Abstract

Magnesium alloys are widely employed in several industrial domains for their outstanding properties. They have a high strength-weight ratio, with a density that is lower than aluminum (33% less), and feature good thermal properties, dimensional stability, and damping characteristics. However, they are vulnerable to oxidation and erosion-corrosion phenomena when applied in harsh service conditions. To avoid the degradation of magnesium, several coating methods have been presented in the literature; however, all of them deal with drawbacks that limit their application in an industrial environment, such as environmental pollution, toxicity of the coating materials, and high cost of the necessary machinery. In this work, a plating of Al_2_O_3_ film on a magnesium alloy realized by the fluidized bed (FB) technique and using alumina powder is proposed. The film growth obtained through this cold deposition process is analyzed, investigating the morphology as well as tribological and mechanical features and corrosion behavior of the plated samples. The resulting Al_2_O_3_ coatings show consistent improvement of the tribological and anti-corrosive performance of the magnesium alloy.

## 1. Introduction

Magnesium, one of the most abundant elements on earth, has an outstanding strength-weight ratio. Their low density, which is about 2/3 that of aluminum, and high specific stiffness make magnesium alloys the lightest structural materials known so far [1]. These properties, in addition to good castability, high thermal and electrical conductivity, high dimensional stability, electromagnetic shielding feature, high damping characteristic, biocompatibility, and high recyclability, make this material extremely valuable in different fields [2]. The most exploited uses of magnesium alloys include automotive and aerospace applications, where the weight-saving feature leads to fuel consumption and CO_2_ emission reduction [3], in addition to biomedical applications [4,5].

The hardest restrictions on the application of magnesium, especially in outdoor background or harsh service conditions, are due to its poor corrosion and wear resistance. The corrosion behavior of magnesium alloys can be enhanced by reducing the heavy metal impurities or controlling the environmental factors. As impurity reduction not economically convenient and environmental modification is not always possible, the most adequate way to overcome the aforementioned drawbacks is to produce a coating for the alloy. However, magnesium is marked by an extreme chemical reactivity, which could be a limitation to most of the coating technologies known to date. As such, the coating produced must be pore free; indeed, the electrochemical potential of magnesium is extremely low and contact with a nobler potential would induce galvanic corrosion, increasing the corrosion rate [6]. The literature offers a great number of possible coating technologies, such as electrochemical plating, conversion coatings, and gas-phase processes [6,7].

Taking into account the cost and feasibility, the electrochemical techniques represent the most effective way to obtain metallic coatings on magnesium-based substrates. Still, particular attention must be paid to the plating baths, since magnesium has a violent reaction when it comes into contact with acid solutions. A Cu-Ni-Cr film showed good corrosion resistance in an interior and mild environment [8]. Further works where good corrosion resistance was obtained by means of electrochemical plating are reported in the literature [9,10,11]. The problems with electroplating and electroless plating include the great consumption of electricity, involving a significant increase of process cost, and the short lifetime of the plating bath, thus raising environmental concerns.

Another way to generate an insulating barrier on a magnesium surface to enhance the corrosion behavior is represented by conversion coatings. The literature presents different types of conversion films such as chromate, phosphate [12,13,14], and anodizing films [15]. The main disadvantages of these techniques are represented by environmental pollution and high toxicity caused by the chemical compounds of the conversion solutions. 

In terms of environmental impact, the gas-phase deposition methods are by far the optimal solutions, even considering the potential emission of hazardous particles. Chemical vapor deposition and physical vapor deposition processes allow laying down several kinds of materials and have already been carried out in several works with the aim of enhancing magnesium features [16,17]. These processes are minimally influenced by the workpiece material and do not require pre-treatments processes. However, the high cost of the machinery and the energy consumption, in addition to the employment of expert operators, exclude these techniques from application in industrial environments.

Functional and theoretical aspects of fluidized bed (FB) technology have been studied and presented in the literature [18,19], as well as several applications of this technique. In the FB deposition process the substrate is placed inside a circular column containing the macroparticles that will compose the coating. The particles are suspended by means of flowing air and behave like a fluid, brushing the whole sample surface. In consequence of the impact, the particles are embedded in the sample surface and the progressive accumulation generates the coating. The maximum thickness reachable by means of this deposition process and the growth rate depend on the substrate material, which is supposed to be softer than the coating in order to ameliorate the processing conditions. Moreover, the grow rate decreases with the treatment time, since the particles accumulation increases the superficial hardness and does not allow the further deposition of particles. The particles that compose the coating must have macrometric dimensions and feature high hardness and brittleness in order to create fragments that will be embedded as a result of the impact with the substrate. The drawback of this process is characterized by the limited geometries it can plate; indeed, the impact of the coating particles must be orthogonal to the sample surface, otherwise the impact will result in the removal of the substrate material.

In comparison with other coating technologies, the FB technique represents an improvement in terms of environmental impact. Indeed, the coating particles do not pour out from the deposition column, avoiding dispersion in the environment, and may be employed for several coating processes.

This paper proposes the production of Al_2_O_3_ film on AZ91 magnesium alloy by means of the FB technique, considering different rotation frequencies and treatment times. Aluminum oxide and ceramics in general are some of the most used materials for hard layers production in several metallic substrates because of their wear and corrosion resistance features, as well as their thermo-mechanical, chemical, and electrical characteristics.

The results obtained show how the process implemented is capable of producing hard and wear-resistant coatings, if precise operational conditions are respected, improving the corrosion behavior of the alloy in comparison to the untreated substrate.

## 2. Materials and Methods

### 2.1. Materials

The dimensions of the magnesium sample, which composition is reported in Table 1, were 18 × 18 mm^2^ for a thickness of 2 mm. The alumina powder used for the experimental procedures (provided by Smyris Abrasivi Srl), the same as that exploited in Reference [20], was characterized by a granulometry with a mesh of 16 for an average diameter dimension of 1.2 mm and a shape factor of 0.67, which is defined as the ratio between the minimum and maximum diameter of the particles.

Each sample was ground with abrasive paper in several steps, with a granulometry up to a grit size of 2500, in order to remove impurities and defects caused by the previous polishing steps.

### 2.2. Equipment and Experimental Test

**Figure 1:**
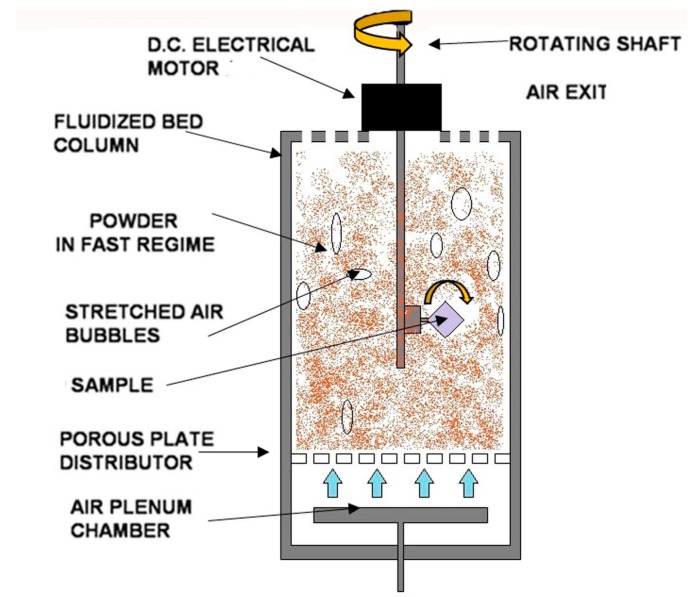
Representation of the fluidized bed (FB) device.

The system used for the FB deposition is schematically reported in Figure 1.

The FB device is a cylindrical column with a section of 6936 mm^2^. A porous bronze plate, the function of which is to homogeneously distribute the flowing air, is placed on the bottom. The pores have a size of 30 µm in order to support the alumina powder when the air is not flowing. Above the porous plate, there is the fluidization column. When the gas is flowing, the particles are suspended in the flow, behaving like a fluid.

The alumina deposition occurs due to the impact of the powder with the sample when the sample rotates. When the impacts occur, the Al_2_O_3_ particles are subjected to fragmentation because of the speed of the rotating sample and their own brittleness. The fragments from the alumina particles are embedded on the substrate surface and the film formation occurs due to their progressive accumulation on the sample surface. As the alumina particles are harder than the magnesium, the grow rate of the coating decreases with treatment time increments. Indeed, once a thick film is formed, the alumina particles cannot be embedded on the sample surface anymore because of the hard layer plated on the substrate; this behavior is known as the “embedding phenomenon” [20].

The experimentation investigated the influence of the frequency of the rotating sample and treatment time on AZ91 for a FB deposition process in an air atmosphere. The samples were kept at ambient temperature. The variables considered for the experimental tests are the rotation frequency of the samples and the treatment duration; in particular, the frequencies chosen were 20, 30, and 40 Hz, while the treatment time was respectively 10, 4, and 4 h. In Table 2, the tangential speed as a function of the frequency is reported, where the minimum speed refers to the velocity of the sample at the point closest to the rotating shaft and the maximum speed refers to the velocity of the sample at the external edge.

The frequencies were chosen as a result of the preliminary test aimed to define the range within which the magnesium is processable. Reduced variation of the rotation frequency was sufficient to lead the process from the embedding phenomena to the micro-cutting, since the hardness of the sample is low, so resultant range is narrow. Considering the lowest frequency, extended process time is required; in order to reduce it, the speed of the sample was increased respectively to 30 and 40 Hz.

The fluidization pressure was imposed to 2 bar, the minimum value that guarantees the particles suspension.

Given that the peripheral velocity of the sample depends on its distance from the rotating axis, during the FB deposition the sample was rotated 90° every hour on an axis orthogonal to the sample surface. This led to a homogeneous deposition throughout the treatment thanks to a homogeneous impact speed on the sample surface.

### 2.3. Characterization Test

The film obtained by means of FB treatment was investigated from several points of view, in order to understand how the parameters chosen affect the film properties.

In order to measure the layer thickness, sections of the samples were observed by SEM microscopy and analyzed with the EDS (Energy Dispersive Spectrometry) technique. Furthermore, the morphology of the plated films was first investigated by stereoscopic images.

At a later stage, the sample roughness was explored with a roughness tester “Taylor-Hobson TalySurf CLI 2000” (Berwyn, PA, USA), evaluating the arithmetic average of height absolute values Ra, the profile maximum height Rz, the average length of profile elements Rsm, and the mean square slope of the profile RΔq.

For the roughness test, a 500 profile was acquired, with a length of 8 mm and a spacing of 1 µm. The measurement speed was 1 mm/s. 

The device employed is capable of producing a three-dimensional (3D) map of the investigated sample. The parameters for the creation of the 3D maps are presented in Table 3.

In order to analyze the hardness of the coating, an indentation test was performed on the exterior surface of the samples. The Vickers micro-hardness value was estimated with a “Micro Combi Tester CSM” device (CSM, Needham, MA, USA). Two different loads were considered for performing the test, 3 N and 5 N, to better evaluate the coating influence on the hardness. The contact load was 0.03 N; for the test performed at 3 N, the loading rate and the unloading rate were 6 N/min, while for the test performed at 5 N they were 10 N/min.

A tribological evaluation of the layer deposited was implemented by means of a “Tribometro CSM” tribometer in order to estimate the wear resistance of the plated film.

A dry-sliding linear reciprocating test was carried out on the samples produced, using a 100Cr6 ball with a diameter of 6 mm as a counterpart, at a sliding speed of 8 cm/s. The wear volume was measured at several sliding distances; in particular, the wear volume evaluation was realized after 5, 10, 20, 30, 50, 70, 100, and 150 m. The wear trail was 3D mapped with a resolution of 1 µm × 2 µm and the removed volume evaluated by means of threshold methods. For each experiment, one sample was tested.

Finally, a corrosion test was performed on the samples surface aimed at evaluating the protection offered by the alumina coating on the magnesium samples. In order to study the corrosion behavior, the “VoltaLab” potenziostat was employed to produce the Tafel curves of the different coatings produced via FB treatment.

The measurement was carried out by means of a three-electrode cell. The sample act as a working electrode, while the counter electrode was made of platinum and the reference electrode was a saturated calomel electrode filled with a KCl solution. Its potential with respect to the SHE (Standard Hydrogen Electrode) was E = 0.412 V.

The corrosion resistance was evaluated in an alkaline solution with 3% NaCl. The first step concerned the sample passivation, imposing a potential of −1500 mV for 120 s. Then, in order to obtain the Tafel curves, the anodic polarization curves was revealed by varying the potential between −1700 and 500 mV, with steps of 2 mV/s. A single corrosion test was performed for each deposition frequency.

## 3. Results and Discussion

**Figure 2:**
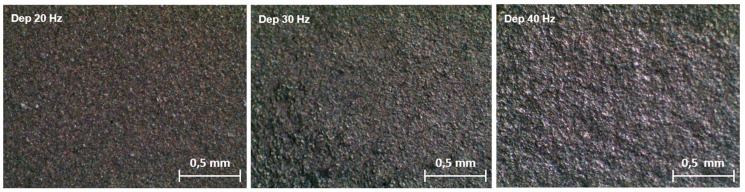
Stereoscope images of films formed at 20 Hz, 30 Hz, and 40 Hz.

Stereoscopic images of the samples reported in Figure 2 show that the substrates coated at 20 and 30 Hz feature a homogeneous deposition. In contrast, the sample produced at 40 Hz is characterized by an indented surface with tiny uncovered spots, as illustrated by the clearer dots on the sample surface.

**Figure 3:**
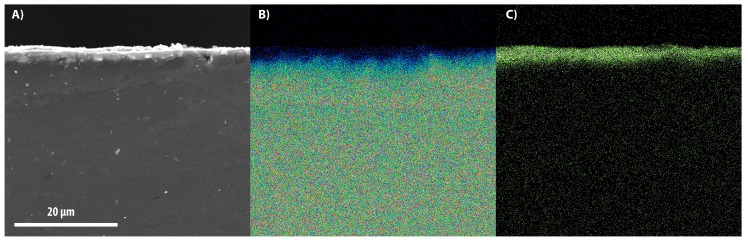
SEM image of the alumina layer (**A**) coated at 20 Hz for 10 h; composition maps of magnesium (**B**) and aluminum (**C**).

**Figure 4:**
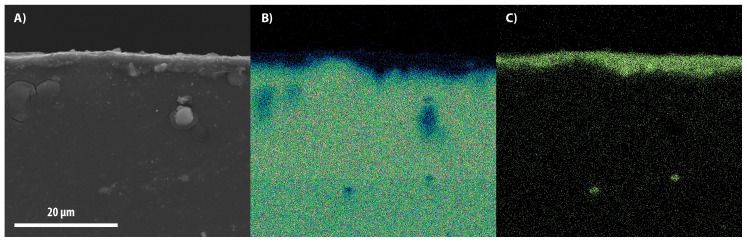
SEM image of the alumina layer (**A**) coated at 30 Hz for 4 h; composition maps of magnesium (**B**) and aluminum (**C**).

**Figure 5:**
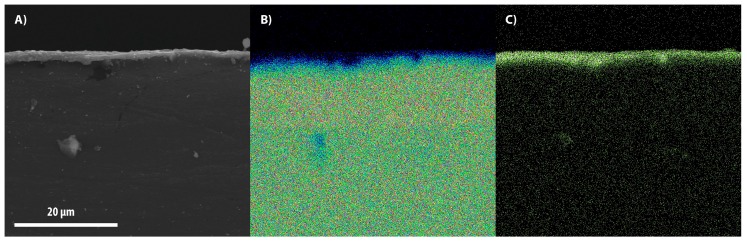
SEM image of the alumina layer (**A**) coated at 40 Hz for 4 h; composition maps of magnesium (**B**) and aluminum (**C**).

**Figure 6:**
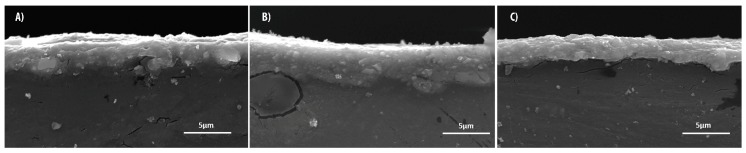
SEM images of sample cross-sections for films formed at (**A**) 20 Hz; (**B**) 30 Hz; (**C**) 40 Hz.

Figure 3, Figure 4 and Figure 5 show the SEM images of the cross-section between the substrates and coatings in panel A, while panels B and C depict the maps of the distribution of magnesium and aluminum contents, respectively. In Figure 6, the coatings are reported with an increased magnification.

The thickness of the layers formed at 20, 30, and 40 Hz are respectively 2.2 ± 0.21 μm, 4 ± 0.36 μm, and 2.3 ± 0.23 μm. The lowest value of the layer thickness was obtained in correspondence with the lowest deposition frequency, despite the long process duration. 

This suggests that the asymptote of the thickness was already reached and the hard superficial layer resulting from the early stage of the process did not allow further accumulation of alumina particles due to the low impact speed. The maximum thickness was obtained with the deposition at 30 Hz. The sample produced at 40 Hz is comparable with the sample at 20 Hz because of the high impact speed of the alumina particles, which entail the localized removal of the coating. It is inferable that the frequency affects the thickness more than the treatment time.

**Figure 7:**
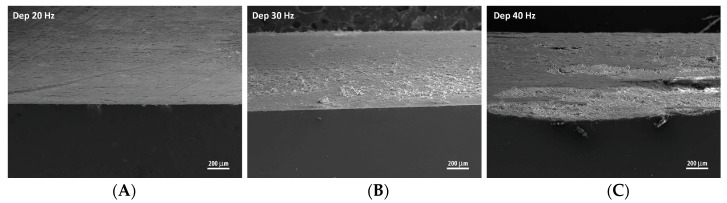
SEM images of the edge of the samples formed at (**A**) 20 Hz; (**B**) 30 Hz; (**C**) 40 Hz.

This behavior is confirmed by the SEM images in Figure 7, acquired by tilting the sample edge in order to observe a portion of the coating of the samples.

The samples coated at 20 and 30 Hz feature a smooth surface, in contrast with the sample coated at 40 Hz. The latter shows hollowed areas as a consequence of the brittle fracture of the superficial layer, caused by the impact with the alumina powder.

**Figure 8:**
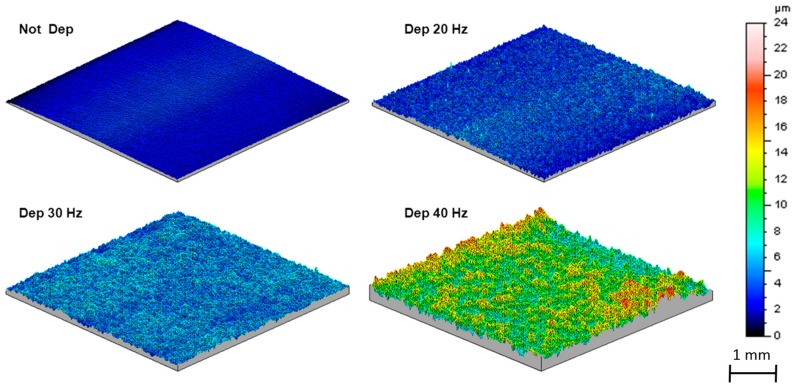
3D superficial maps of samples.

The 3D maps obtained and reported in Figure 8 confirm how the irregularity of the morphology grows with the deposition frequency. 

Particularly, it is evident that the great fluctuation of the profile acquired from the sample formed at 40 Hz is a consequence of the material removal.

**Figure 9:**
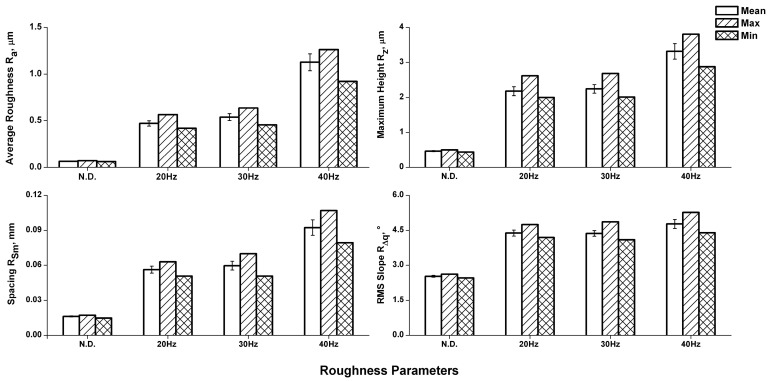
Roughness analysis results.

The roughness analysis, which results are reported in Figure 9, allowed the evaluation of the following parameters: average roughness, maximum height, spacing, and RMS (root mean square) slope.

The roughness parameters confirm the indication obtained from the 3D maps; the initial smooth surface exhibits an increase in the roughness parameter.

The results for the coatings formed at 20 and 30 Hz are comparable, due to the progressive accumulation of the Al_2_O_3_ particles on the sample surface. Instead, the great increase of Ra and Rz observed in the coating formed at 40 Hz is caused by the removal of the coated film.

**Figure 10:**
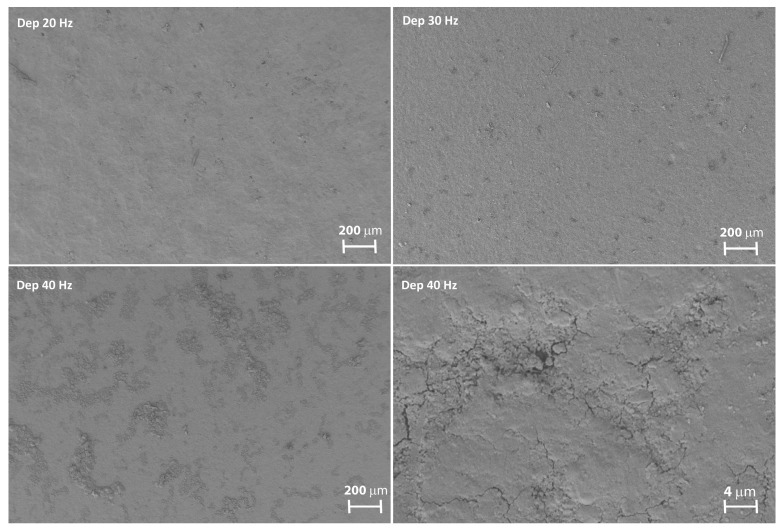
SEM images of the superficial coating layer.

From the SEM images in Figure 10, is deducible that, for the coatings formed at 20 and 30 Hz, homogenous deposition was achieved, while for that formed at 40 Hz there are visible damaged areas. In particular, high frequencies allow the alumina deposition, while extended process time results in the brittle expulsion of material from the plating. 

**Figure 11:**
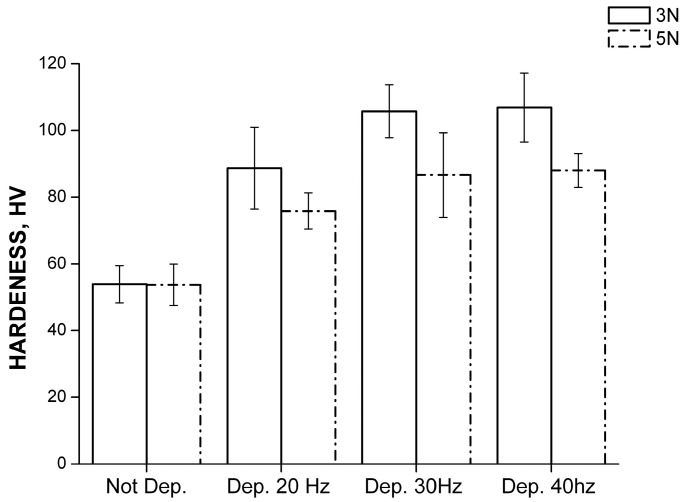
Hardness value obtained as a function of the indenter load and deposition frequency.

The mechanical behavior of the films was then tested. The first investigation implemented was the micro-hardness test. The variables considered during the test were the deposition frequency and the indenter load. The results are shown in Figure 11.

The first evidence is the improvement of the samples’ hardness for all of the frequencies considered. All of the films thus led to a better performance of the substrate.

The result shows how the higher values of hardness are obtained with a lower load applied; the reduced penetration depth of the indenter allowed the analysis of coating, reducing the influence of the substrate on the measurement.

**Figure 12:**
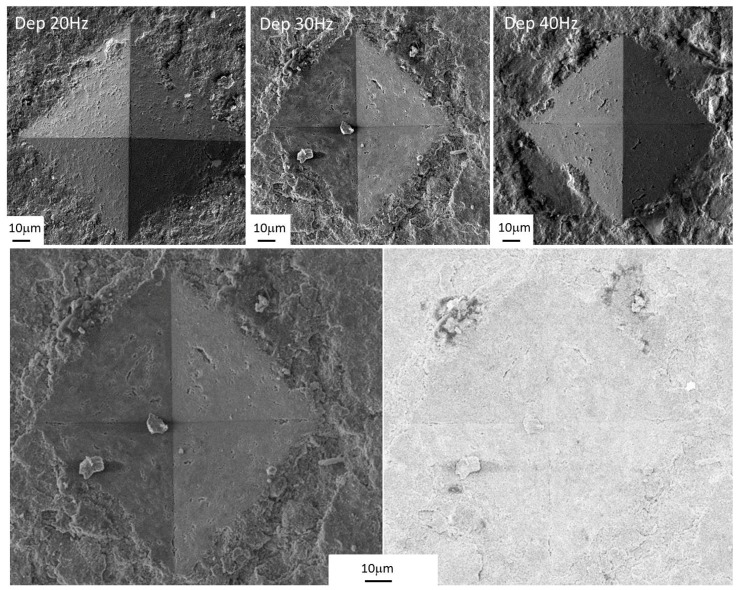
SEM images of the indenter imprint.

For the coatings formed at 30 and 40 Hz, similar values were measured. For the deposition at 30 Hz, this attitude is due to the greater thickness of the layer, whereas at 40 Hz it may be explained by the higher speed of the alumina particles that entail an increased hardening of the substrate. The SEM images of the indenter imprint are reported in Figure 12 and do not highlight pile-up effects. Furthermore, the back-scattering images highlight that no cracking phenomena occurred.

**Figure 13:**
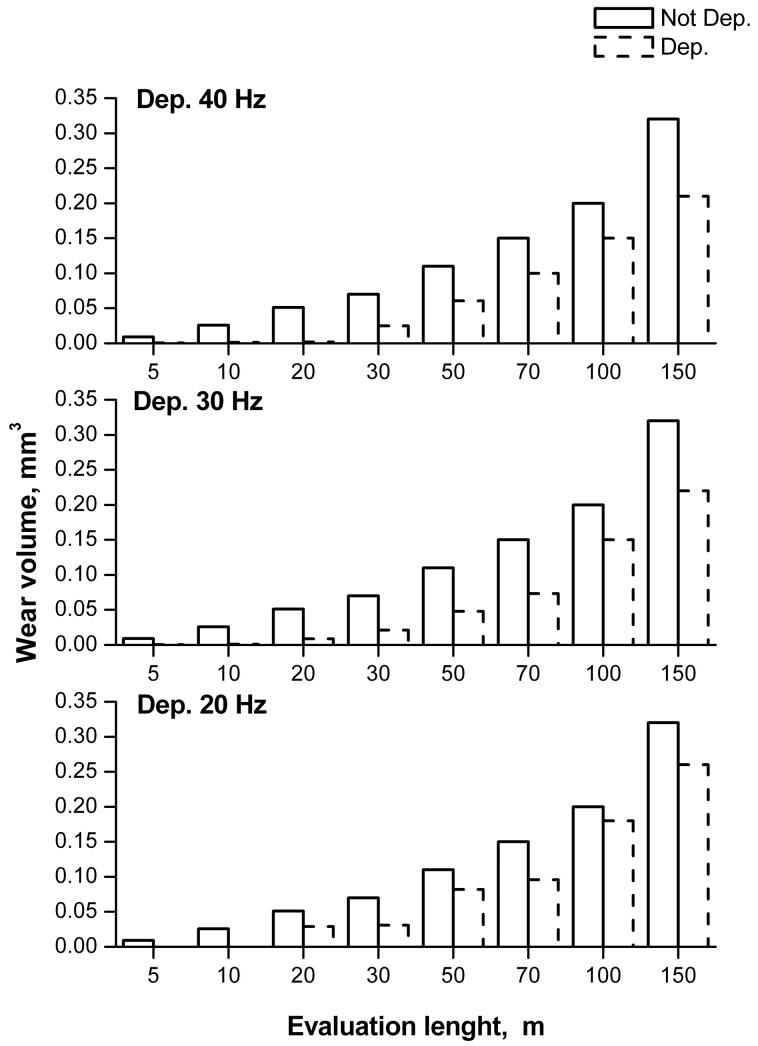
Removed volume function of the distance covered by the indenter.

The wear resistance was then tested and the results are shown in Figure 13.

The first consideration concerns the capability of the FB deposition of alumina to produce a better wear resistance in comparison with the simple magnesium alloy, for all of the frequencies considered. While the samples plated at 30 and 40 Hz present a similar wear volume at the end of the test, the sample coated at 20 Hz exhibited an increment of about 15%. Indeed, at 30 and 40 Hz the greater hardness lead to a wear volume reduction. The crevice and ditch produced by the deposition at 40 Hz do not affect the wear phenomena, featuring a dimension much lower than the counterpart diameter of the ball employed for the test.

Moreover, significant values were obtained in the early stages of the test; after 20 m of test the wear volume for the coatings deposited at 30 and 40 Hz was almost negligible, while that for the coating formed at 20 Hz was found to be 0.029 mm^3^. This behavior is due to the reduced thickness of the plated layer. In fact, a shorter testing period is sufficient to reach the substrate that, being softer than the alumina layer, presents a higher wear rate.

**Figure 14:**
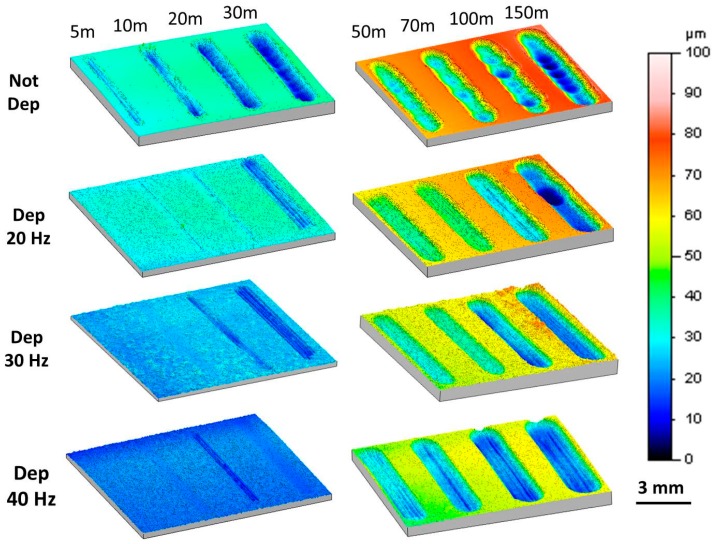
3D maps of the wear tracks.

The 3D maps of the wear tracks are reported in Figure 14. The analysis shows that the wear tracks of the magnesium sample are different from the tracks of the coated substrates. In the coated samples, on the bottom of the groove produced by the counterpart there are scratches that are parallel to the wear track axis, as is typical for the three-body wear mechanism. On the contrary, on the magnesium sample the wear tracks do not highlight scratches but exhibit an irregular bottom. In correspondence to the inversion point of the sliding of the counterpart, the friction coefficient increases, causing adhesion phenomena with the substrate. This behavior is responsible for the dragging of the material in the internal part of the wear track. The progressive accumulation of this material leads to an irregular bottom that implies the bouncing of the counterpart.

**Figure 15:**
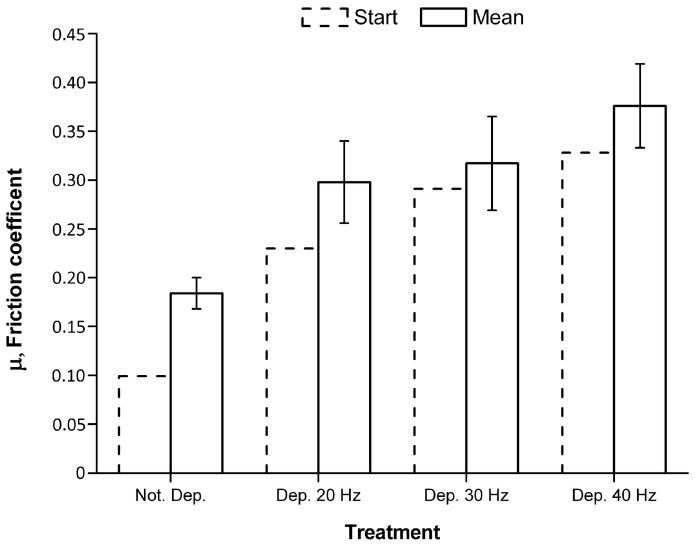
Friction coefficient analysis.

The friction coefficient was then analyzed and the results are reported in Figure 15. As expected in consequence of the roughness results, the samples coated at 20 and 30 Hz present similar results of the friction coefficient, while that coated at 40 Hz shows an increase due to the coating removal and the formation of ditches.

The analysis of the friction coefficient also allowed the determination of when the coating samples were worn off. The results are reported in Table 4.

Despite of the minor thickness in comparison with the sample plated at 30 Hz, the high speed of the particles during the deposition at 40 Hz entail an increased compactness that, along with the hardening of the substrate, led to an increase in the distance covered before the film was worn off.

**Figure 16:**
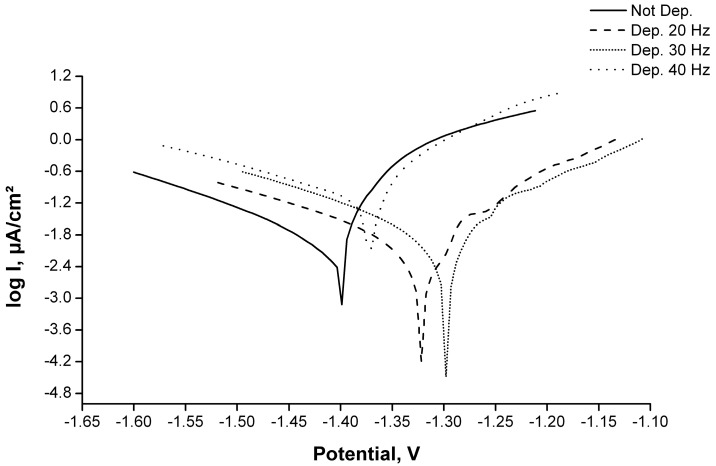
Tafel curves of coated samples.

Finally, the corrosion resistance test was carried out, obtaining the potentiodynamic polarization curves analysis of the samples, as shown in Figure 16. The relative values of the corrosion potential and current (E_corr_ and I_corr_) are reported in Table 5.

Considering the corrosion potential, the best results were obtained for the samples coated at 20 and 30 Hz, with similar results. Although the sample with the coating deposited at 40 Hz also showed a shift of this parameter to a nobler value, this behavior was less significant that of the specimens treated at 20 and 30 Hz. The same trend was observed for the corrosion current, but while the deposition at 20 and 30 Hz led to an improvement of the current density, the sample plated at 40 Hz was found be worse than the untreated sample.

This behavior indicates that for the purpose of corrosion resistance enhancement, the surface morphology plays a more critical role in comparison to the thickness. Indeed, the samples treated at 20 and 30 Hz show similar features in terms of corrosion resistance and surface morphology, even though the thickness is different.

On the contrary, at 40 Hz the high speed of the colliding particles involves the creation of ditches and crevices in the surface, creating a different morphology compared with the other samples investigated. This phenomenon not only affects the barrier efficiency of a coating, but also establishes a galvanic corrosion process and thus a high corrosion rate.

There are several methods presented in the literature that can be compared with the proposed method. Of these, electroplating is one of the most exploited. Generally speaking this kind of application allows reaching a higher superficial thickness within the range of 10–30 µm, about 5 to 15 times larger than that obtained in this work. The increased thickness provides better insulation of the substrate and thus a better corrosion behavior. In particular, the paper presented by Arman Zarebidaki et al. [21] deals with the electrodeposition of a nano-crystalline nickel coating on an AZ91 magnesium substrate. In this paper, a nickel layer was coated after a zincate pre-treatment and a copper undercoating, for a global thickness of 10 µm obtained by a process that lasted 45 min. In this context, their work led to an enhancement of the corrosion potential in a 3.5% NaCl solution, exhibiting an increase from approximately −1.5 V to −1 V.

In the field of conversion coatings, the work of Songlin Mu et al. [22] can be set as basis for comparison. In their work, the authors developed a Mo-Ce conversion coating. The thickness of the obtained layer reached 2–3 µm after 15 min of treatment; however, a longer time resulted in wide crack formation in the coating. The analysis of the corrosion potential, conducted in a 3.5% NaCl solution, showed how it was improved from −1.6 V to −1.35 V for the coated samples.

The vapor phase deposition represents the state of the art in coating technologies. Hoche et al. [23] studied the corrosion behavior of different coatings plated on a magnesium substrate via PVD (physical vapour deposition) deposition. As reported, the thickness obtained varied the function of the layer plated. Considering the coating with a similar thickness to that reported in this work, the TiN and Al_2_O_3_ coatings respectively reached 2.3 and 3 µm in about 2 h of treatment, and the relative corrosion potential was evaluated to be about −1.3 V.

Considering the results achieved in the published literature, the method proposed in this paper leads toward the production of a protective layer with a performance in corrosion protection that is comparable to other technologies, beyond the morphologic difference function of the method employed. Despite the similar performance, fluidized bed deposition is preferable due to the low cost of the process in addition to low environmental impact ensured by the large dimension of the particles, the lack of chemical additives, and the typically closed structure of the fluidized bed device that does not allow the dispersion of hazardous particles.

## 4. Conclusions

A functional coating of alumina particles was plated on magnesium AZ91 samples, enhancing the tribological and corrosion resistance behavior of the substrates.

The results show how the FB technique allows the production of a hard alumina layer on a magnesium-based substrate, capable of improving the overall features of the investigated alloy. The range of the rotation frequency, within which the FB device improves the quality of the deposited film, reaches a maximum value of 30 Hz. After that, the elevated colliding speed entails material removal from the sample surface, resulting in a higher roughness and the formation of crevices on the superficial alumina layers that thus worsen the corrosion resistance behavior. 

Yet, the high impact speed does not worsen the tribological features of the layers plated, as shown by the wear resistance and micro-hardness test. This is due to the small dimensions of the crevices induced by the high rotational speed at 40 Hz in comparison with the dimensions of the counterpart in the wear test. In general, the proposed solution can be considered as a valid alternative in plating technologies.

## Figures and Tables

**Table 1:** AZ91 composition.

Element	Weight %
**Mg**	90.17
**Al**	9.00
**Zn**	0.70
**Mn**	0.13

**Table 2:** Speeds of the samples as a function of rotation frequency and distance from the axis.

Frequency (Hz)	Max Speed (m/s)	Min Speed (m/s)	Mean Speed (m/s)
**20**	1.256	4.396	2.826
**30**	1.884	6.594	4.239
**40**	2.512	8.792	5.652

**Table 3:** Three-dimensional (3D) maps acquisition parameters.

Parameters	Value
**X axis length**	4 mm
**Y axis length**	4 mm
**Spacing X**	2 µm
**Spacing Y**	2 µm
**Measurement speed**	2 mm/s

**Table 4:** Distance covered when the coating was worn off.

**Frequency (Hz)**	20	30	40
**Distance (m)**	14.47	16.74	19.02

**Table 5:** Corrosion potential and corrosion current of the sample treated with the FB process.

Frequency (Hz)	E_0_ (V)	I_corr_ (μA)
**Untreated**	−1.395	5.627
**20**	−1.324	0.013
**30**	−1.298	0.015
**40**	−1.371	29.896
